# Supplementary material for: A noncanonical RNA-binding domain of the fragile X protein, FMRP, elicits translational repression independent of mRNA G-quadruplexes
Source: J Biol Chem. 2022 Oct 31;298(12):102660. doi: 10.1016/j.jbc.2022.102660 (PMC9712993; doi:10.1016/j.jbc.2022.102660)
Supplement: Supporting information [file mmc1.docx]

**SUPPORTING INFORMATION**

**A noncanonical RNA-binding domain of the fragile X protein, FMRP, elicits translational repression independent of mRNA G-quadruplexes**

MaKenzie R. Scarpitti, Julia E. Warrick, Evelyn L. Yoder, Michael G. Kearse

**
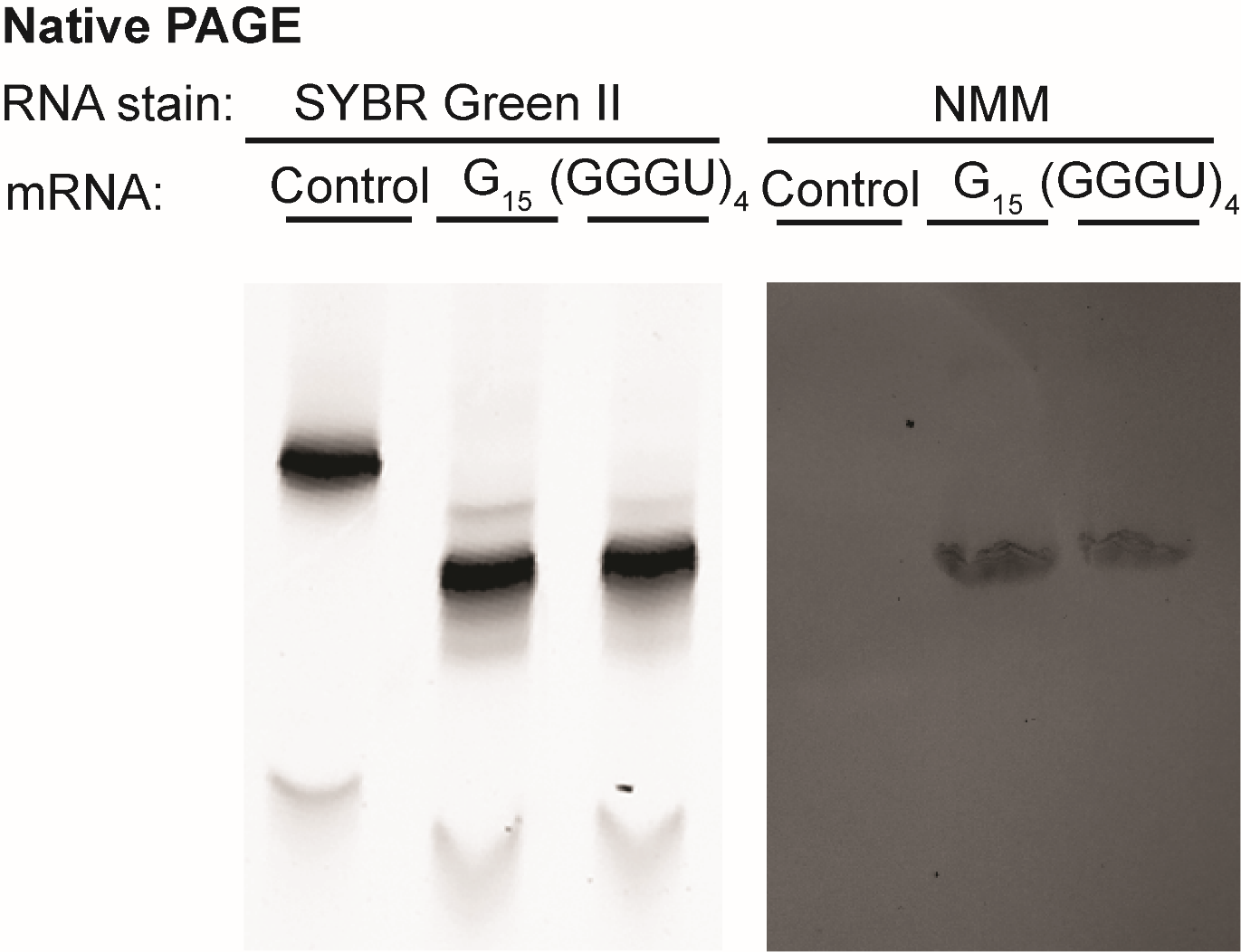
**

**Supplemental Figure S1. G_15_ and (GGGU)_4_** **reporter mRNA, but not control reporter mRNA, harbor G-quadruplexes.** Native PAGE of control, G_15_, and (GGGU)_4_ reporters stained for total RNA with SYBR Green II or for G4 structures with NMM.

**
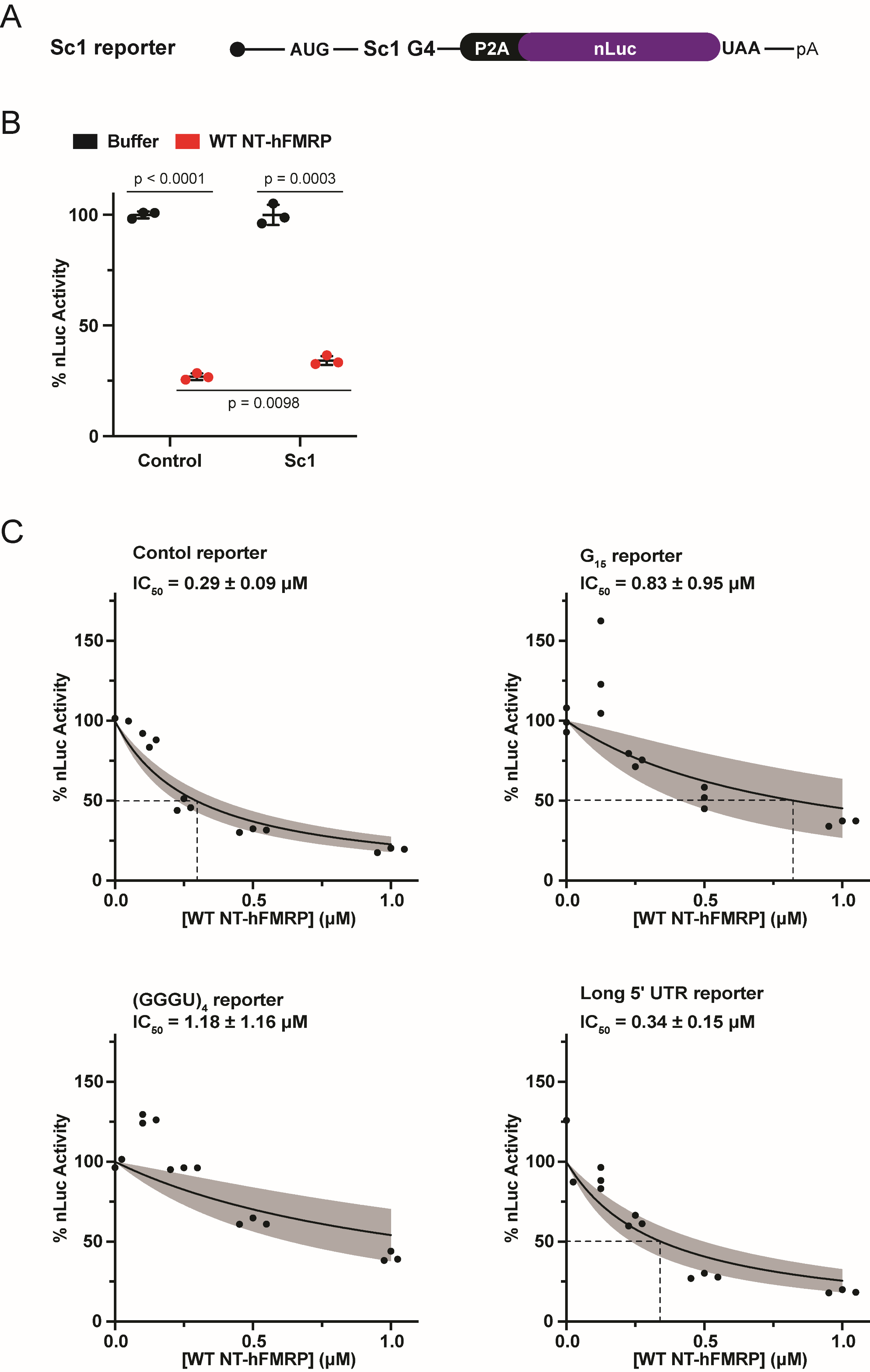
**

**Supplemental Figure S2. The Sc1 G4 does not enhance translational repression by FMRP.** A) Schematic of custom nLuc reporter harboring the Sc1 G4 in the coding sequence. A P2A ribosome skipping motif was included immediately upstream of the nLuc coding sequence to ensure equal nLuc function between reporters. B) *In vitro* translation of Sc1 reporter mRNA with protein storage buffer or WT NT-hFMRP. Data are shown as mean ± SD. n = 3 biological replicates. Comparisons were made using an unpaired t test with Welch’s correction. C) *In vitro* translation of control, G_15_, (GGGU)_4_, and long 5ʹ UTR nLuc mRNA with a titration of recombinant wildtype NT-hFMRP. IC_50_ values were determined for control nLuc (0.29 ± 0.09 µM), G_15_ nLuc (0.83 ± 0.95 µM), (GGGU)_4_ nLuc (1.18 ± 1.16 µM), and long 5ʹ UTR nLuc (0.34 ± 0.15 µM) reporter mRNAs. n=3 biological replicates. A non-linear regression was used to calculate the IC_50_ and is shown as the line with the 95% confidence interval (CI) included as a watermark. The IC_50_ is reported ± 95% CI.

**
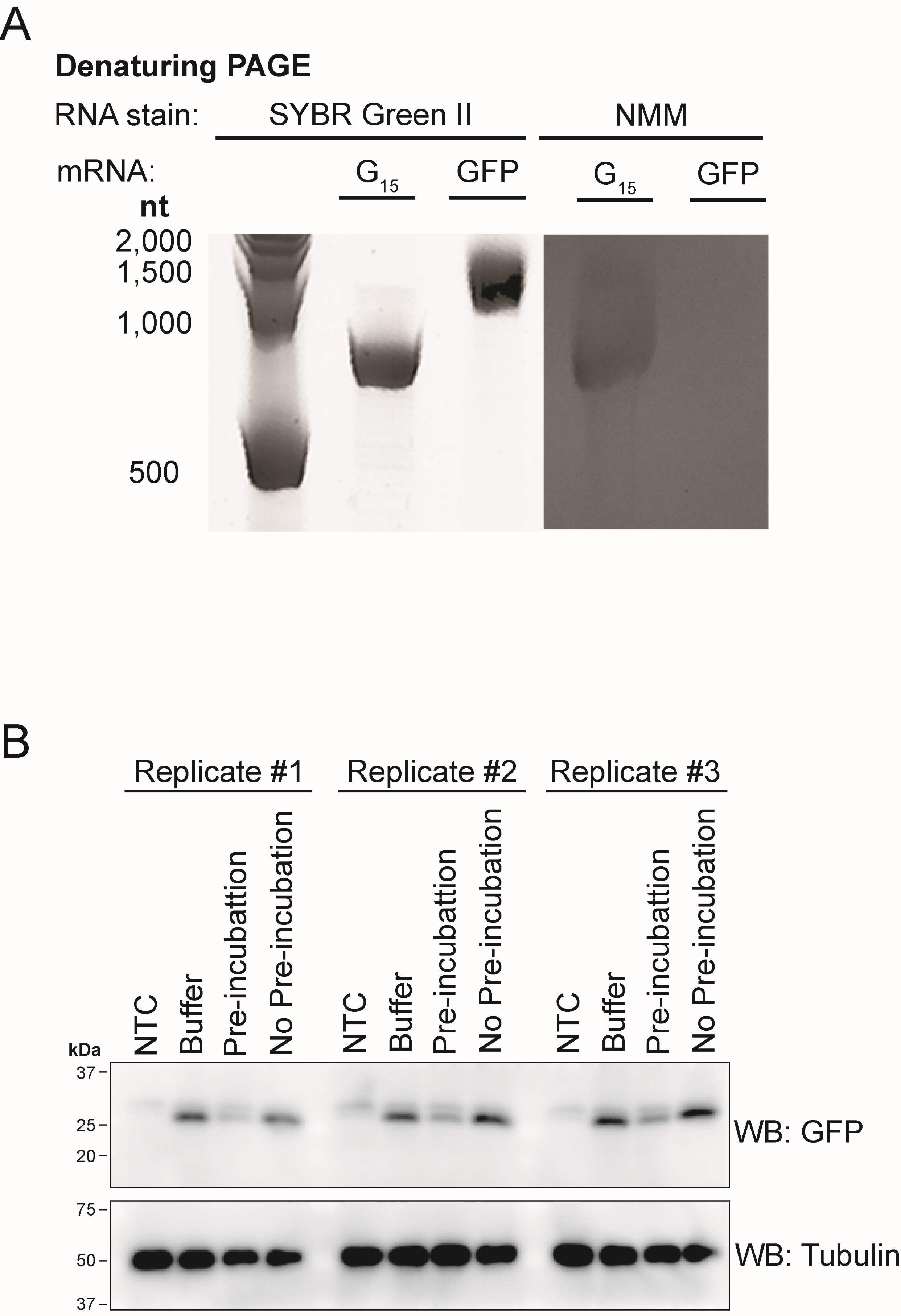
**

**Supplemental Figure S3. NT-hFMRP inhibits translation of G4-less mEGFP mRNA.** A) Denaturing PAGE of G_15_ nLuc reporter and mEGFP mRNAs stained for total RNA with SYBR Green II or for G4 structures with NMM. B) Anti-GFP Western blot of *in vitro* translation reactions of G4-less mEGFP reporter mRNA with an no template control (NTC), protein storage buffer as an additional negative control, with 1 µM WT NT-hFMRP and G4-less mEGFP mRNA pre-incubated together, and with 1 µM WT NT-hFMRP and G4-less mEGFP without a pre-incubation step. Tubulin was used as a loading control.

**
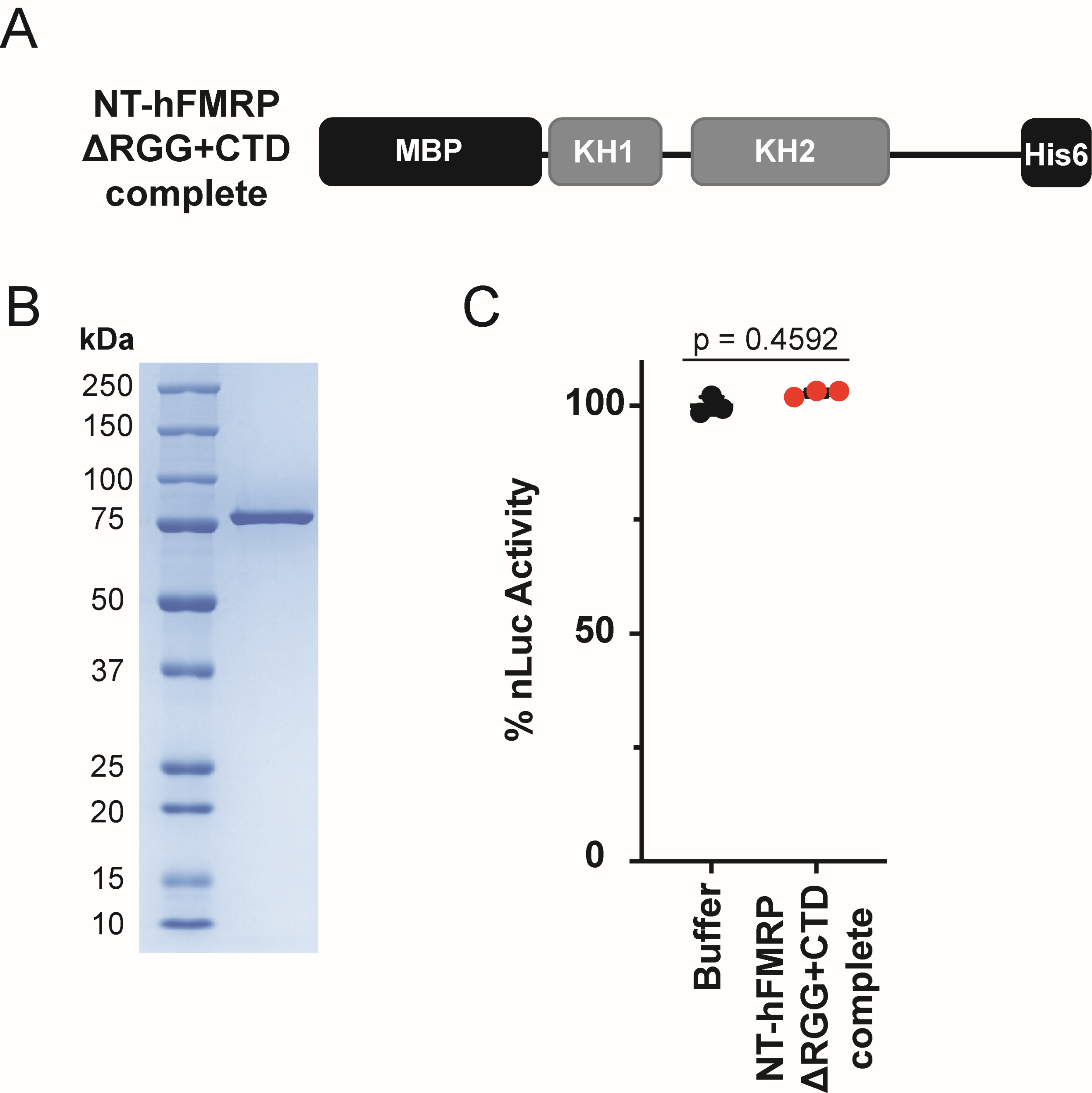
**

**Supplemental Figure S4. NT-hFMRP ΔRGG+CTD complete does not inhibit translation.** A) Schematic of MBP- and His6-tagged NT-hFMRP ΔRGG+CTD complete. B) Coomassie stain of recombinant NT-hFMRP ΔRGG+CTD complete. C) *In vitro* translation of nLuc control mRNA with protein storage buffer or NT-hFMRP ΔRGG+CTD complete. Data are shown as mean ± SD. n = 3 biological replicates. Comparisons were made using an unpaired t test with Welch’s correction.


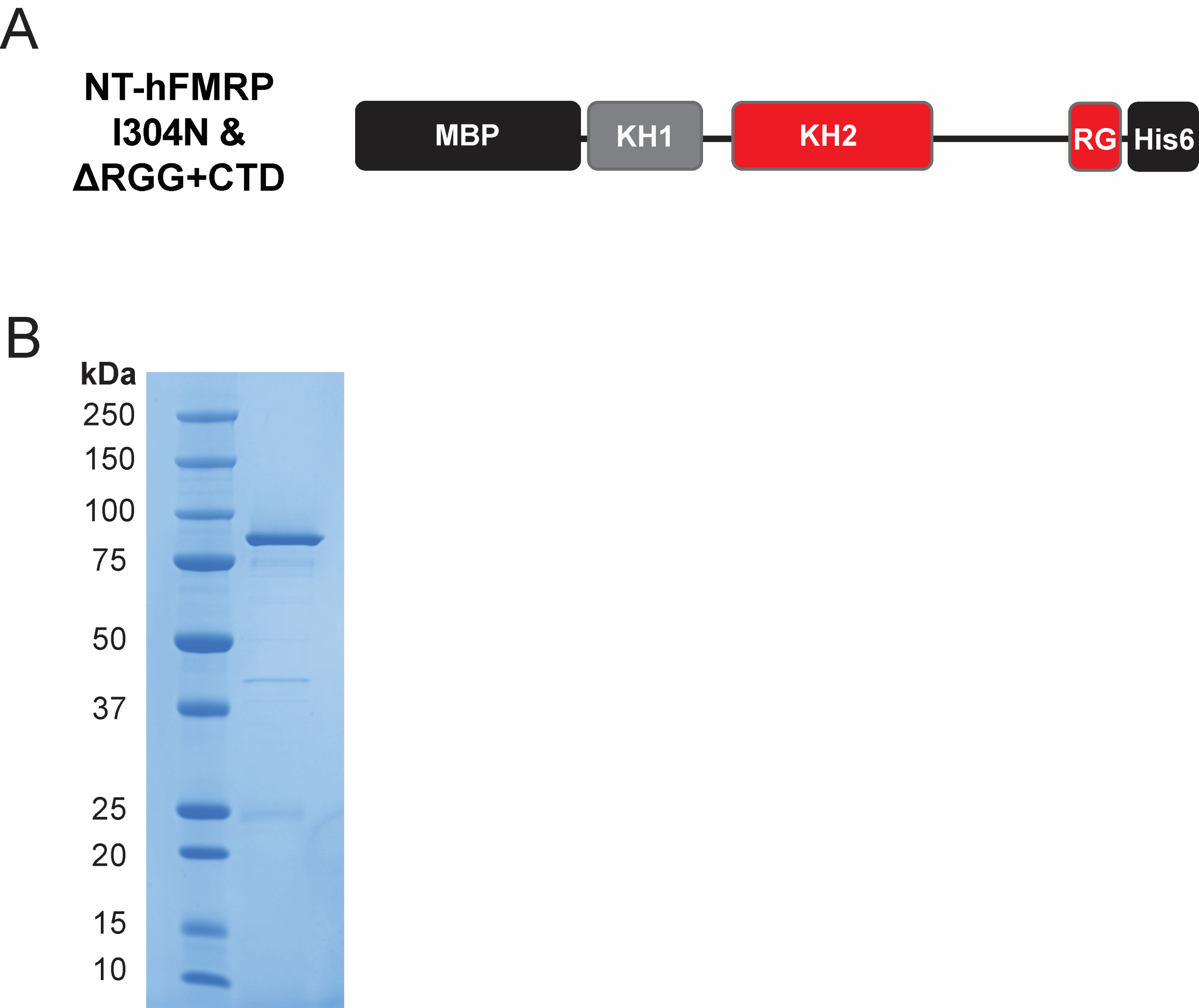


**Supplemental Figure S5. NT-hFMRP I304N & ΔRGG+CTD double mutant.** A) Schematic of MBP- and His6-tagged NT-hFMRP I304N & ΔRGG+CTD. Mutated/truncated domains are highlighted in red. B) Coomassie stain of recombinant NT-hFMRP I304N & ΔRGG+CTD.


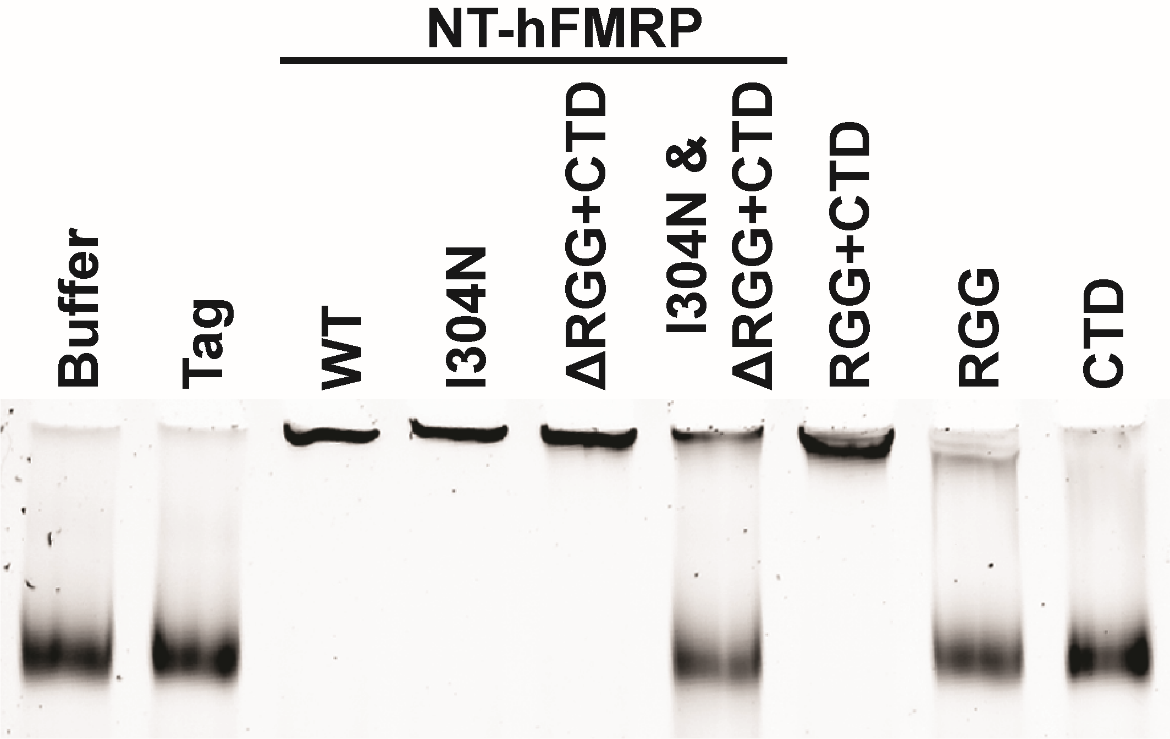


**Supplemental Figure S6. The RGG+CTD ncRBD binds G4-less mEGFP mRNA**. EMSA of G4-less mEGFP mRNA with the indicated recombinant protein resolved on a native PAGE and subsequently stained with SYBR Green II.

**
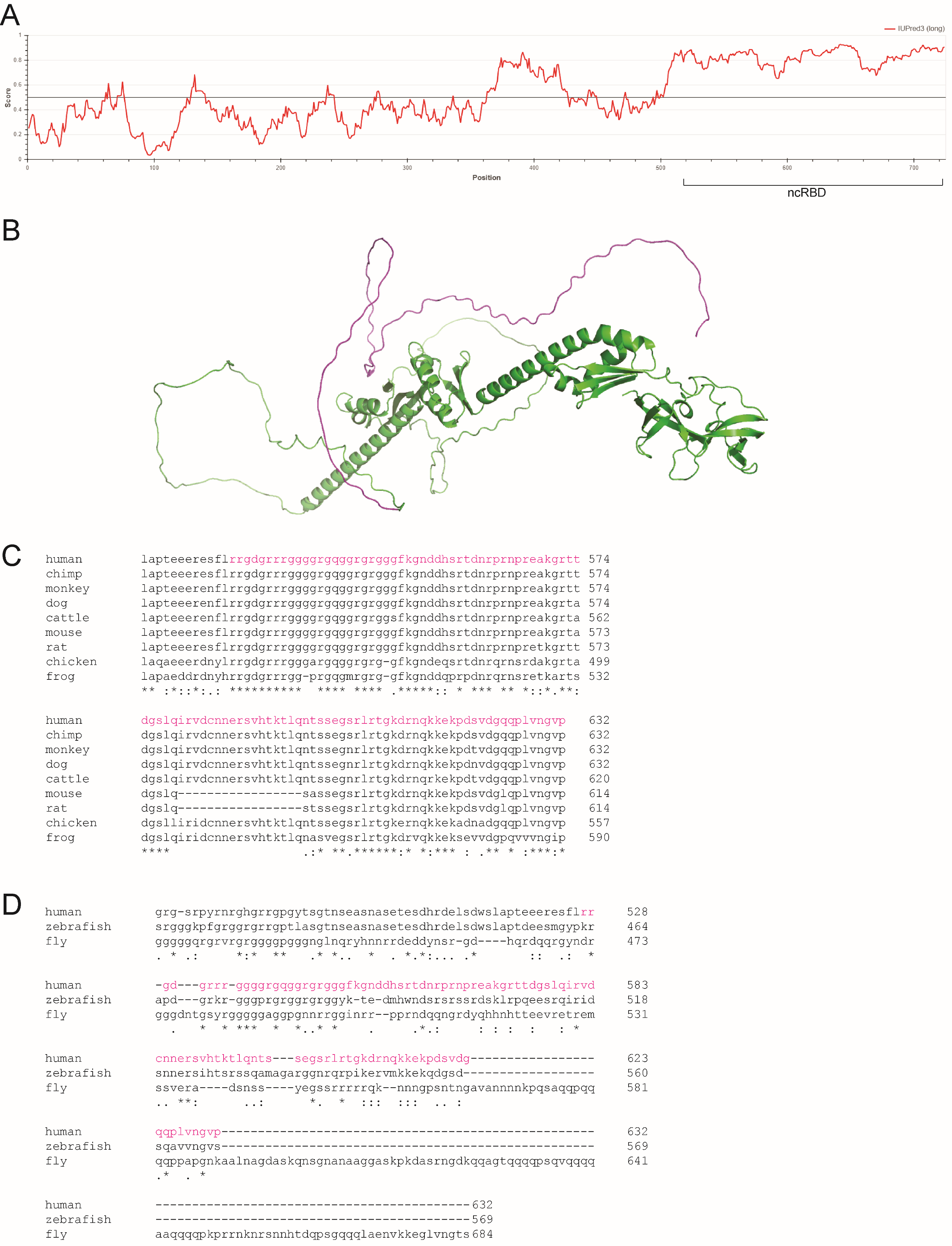
**

**Supplemental Figure S7. The FMRP ncRBD is predicted to be flexible and is conserved among most vertebrates.** A) IUPred3 analysis (<https://iupred.elte.hu/>) of the full-length human FMRP. The ncRBD is bracketed. Scores above 0.5 (solid horizontal line) signal predicted unstructured flexible regions. B) AlphaFold structural prediction of full-length human FMRP with the ncRBD highlighted in magenta. C) Clustal Omega (1.2.4) alignment of vertebrate FMRP orthologs. The human FMRP ncRBD is highlighted in magenta. The residue numbering corresponds to the full-length protein sequence. D) Clustal Omega (1.2.4) alignment of human, zebrafish, and fruit fly FMRP. The human FMRP ncRBD is highlighted in magenta. The residue numbering corresponds to the full-length protein sequence.


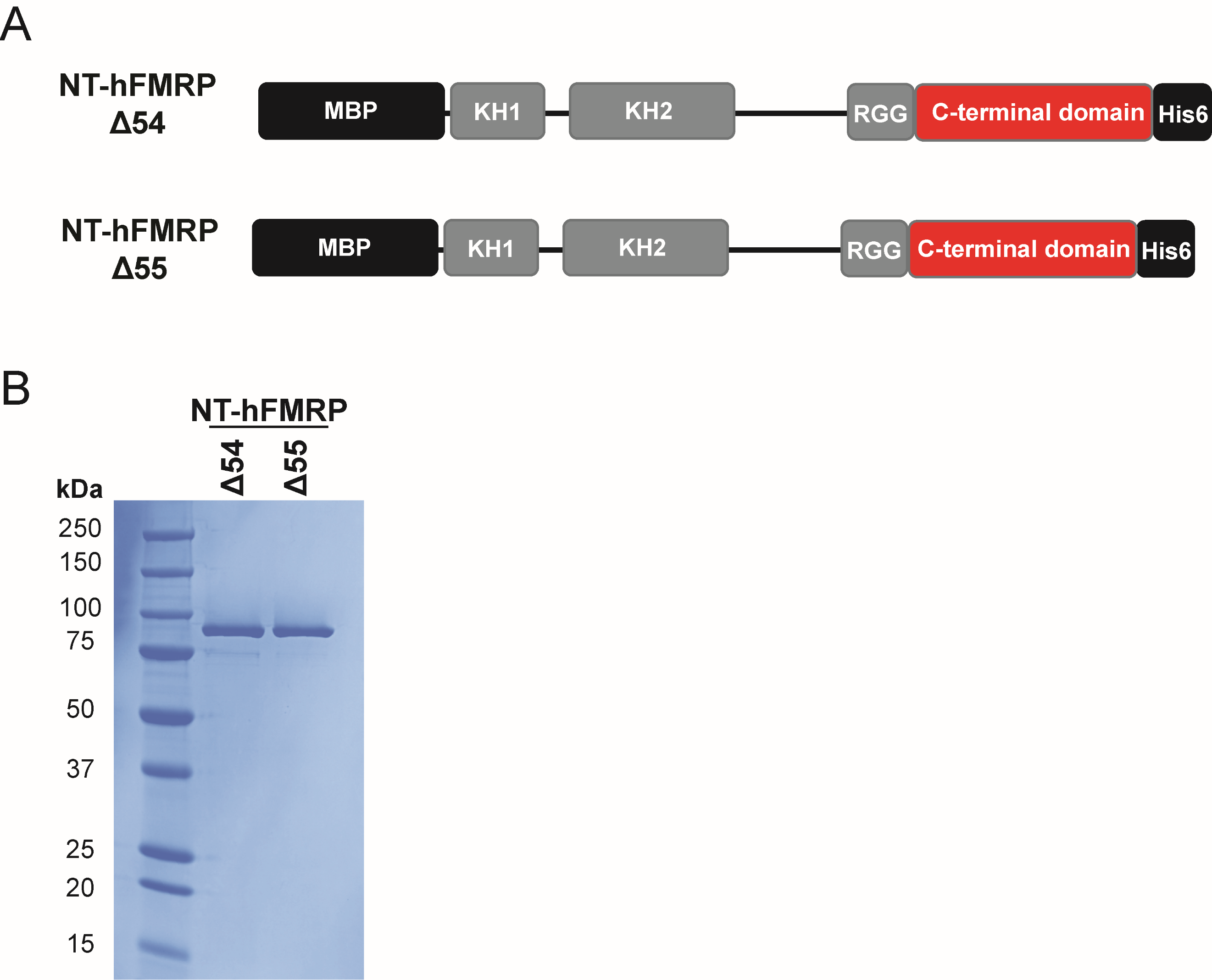


**Supplemental Figure S8. NT-hFMRP Δ54 and Δ55.** A) Schematic of MBP- and His6-tagged NT-hFMRP Δ54 and Δ55. The truncated CTD is highlighted in red. B) Coomassie stain of recombinant NT-hFMRP Δ54 and Δ55.


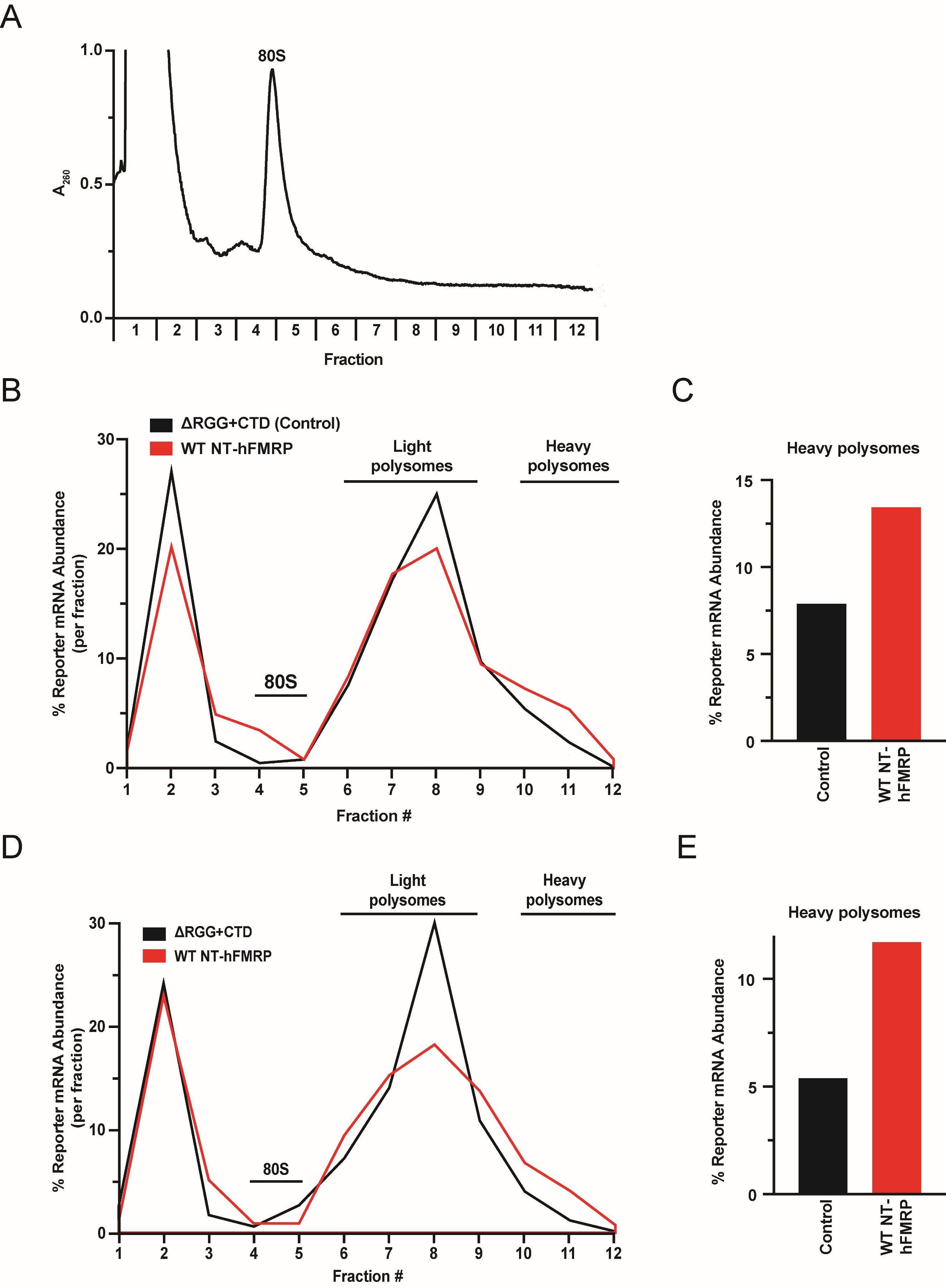


**Supplemental Figure S9. WT NT-hFMRP causes heavy polysomes to accumulate.** A) A representative A_260 nm_ trace of sucrose gradient ultracentrifugation (polysome analysis) of *in vitro* translation reactions. The 80S monosome sediments in fractions 4 and 5. Thus, fractions 6-12 contain polysomes. Robust polysome curves are not visualized as mRNA input amounts were limited to ensure responses were in the linear dynamic range (see Experimental Procedures). B-E) Separate biological replicates of experiments shown in **Figure 6B-D**. B & D) Distribution of reporter mRNA across sucrose gradients to assess polysome formation with ΔRGG+CTD (Control) or WT NT-hFMRP. Abundance of reporter mRNA in each gradient fraction was determined by RT-qPCR. C & E) Cumulative nLuc abundance in heavy polysomes in fractions 10-12 from ΔRGG+CTD (Control) and WT NT-hFMRP samples.


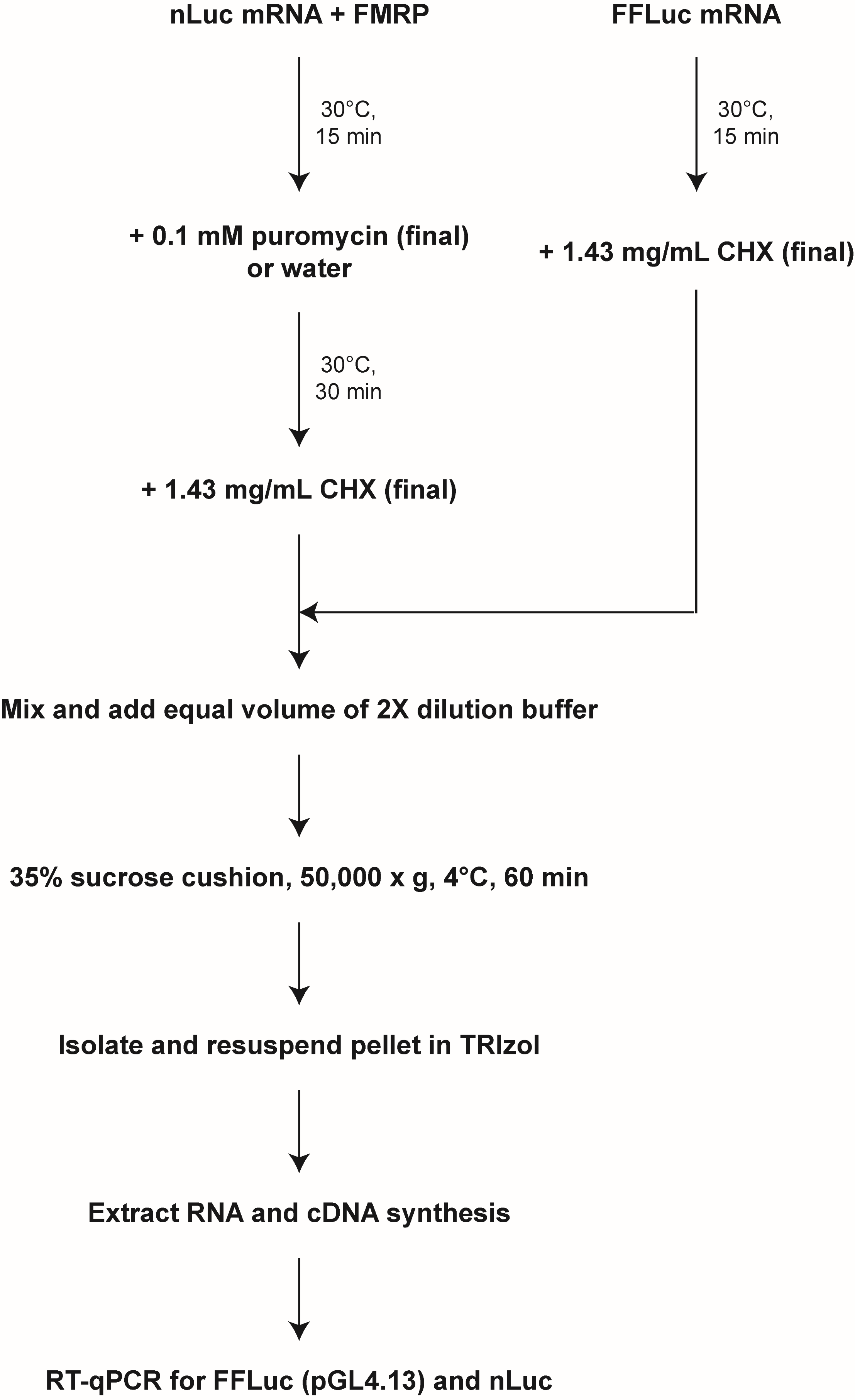


**Supplemental Figure S10. Workflow of puromycin-mediated ribosome dissociation and low-speed sucrose cushion assay.** WT or mutant NT-hFMRP was allowed to form an mRNP with nLuc mRNA as described in the Experimental Procedures. FFLuc (pGL4.13) mRNA was translated and used as an internal spike-in control for ribosome-bound mRNA pelleting and RT-qPCR. See details in Experimental Procedures.

**Reporter Sequences**

*T7 promoter*

Human beta globin 5ʹ UTR

**Heterologous 5ʹ UTR**

Start codon

P2A ribosome skipping motif

nLuc

3xFLAG

Stop codon

G4 insert

**Control nLuc reporter**

*TAATACGACTCACTATAG*GGACATTTGCTTCTGACACAACTGTGTTCACTAGCAACCTCAAACAGACACCATGGGAAGCGGAGCTACTAACTTCAGCCTGCTGAAGCAaGCTGGAGACGTcGAaGAaAACCCTGGACCTGTGTTTACCCTtGAAGATTTTGTcGGCGATTGGCGCCAGACCGCGcGCTATAACCTcGATCAaGTGCTcGAACAaGGCGGCGTGAGCAGCCTGTTTCAGAACCTGGGCGTGAGCGTGACCCCGATTCAGCGCATTGTGCTGAGCGGCGAAAACGGCCTGAAAATTGATATTCATGTGATTATTCCGTATGAAGGCCTGAGCGGCGATCAGATGGGCCAGATTGAAAAAATTTTTAAAGTcGTaTATCCtGTcGATGATCATCATTTTAAAGTGATTCTGCATTATGGCACCCTGGTGATTGATGGCGTGACCCCGAACATGATTGATTATTTTGGCCGCCCGTATGAAGGCATTGCtGTtTTTGATGGCAAgAAgATTACCGTGACCGGCACCCTGTGGAACGGCAACAAAATTATTGATGAACGCCTGATTAACCCaGATGGCAGCCTGCTGTTTCGCGTGACCATTAACGGCGTGACCGGCTGGCGCCTGTGCGAACGCATTCTcGCaGACTACAAAGACCATGACGGTGATTATAAAGATCATGACATCGATTACAAGGATGACGATGACAAGTAAGGCCGCGACTCTAGAG

**G_15_ nLuc reporter**

*TAATACGACTCACTATAG*GGACATTTGCTTCTGACACAACTGTGTTCACTAGCAACCTCAAACAGACACCATGCAACAACAACAACAAGGGGGGGGGGGGGGGCAACAACAACAACAAGGAAGCGGAGCTACTAACTTCAGCCTGCTGAAGCAaGCTGGAGACGTcGAaGAaAACCCTGGACCTGTGTTTACCCTtGAAGATTTTGTcGGCGATTGGCGCCAGACCGCGcGCTATAACCTcGATCAaGTGCTcGAACAaGGCGGCGTGAGCAGCCTGTTTCAGAACCTGGGCGTGAGCGTGACCCCGATTCAGCGCATTGTGCTGAGCGGCGAAAACGGCCTGAAAATTGATATTCATGTGATTATTCCGTATGAAGGCCTGAGCGGCGATCAGATGGGCCAGATTGAAAAAATTTTTAAAGTcGTaTATCCtGTcGATGATCATCATTTTAAAGTGATTCTGCATTATGGCACCCTGGTGATTGATGGCGTGACCCCGAACATGATTGATTATTTTGGCCGCCCGTATGAAGGCATTGCtGTtTTTGATGGCAAgAAgATTACCGTGACCGGCACCCTGTGGAACGGCAACAAAATTATTGATGAACGCCTGATTAACCCaGATGGCAGCCTGCTGTTTCGCGTGACCATTAACGGCGTGACCGGCTGGCGCCTGTGCGAACGCATTCTcGCaGACTACAAAGACCATGACGGTGATTATAAAGATCATGACATCGATTACAAGGATGACGATGACAAGTAAGGCCGCGACTCTAGAG

**(GGGU)_4_ nLuc reporter**

*TAATACGACTCACTATAG*GGACATTTGCTTCTGACACAACTGTGTTCACTAGCAACCTCAAACAGACACCATGCAACAACAACAACAAGGGTGGGTGGGTGGGTTTCAACAACAACAACAAGGAAGCGGAGCTACTAACTTCAGCCTGCTGAAGCAaGCTGGAGACGTcGAaGAaAACCCTGGACCTGTGTTTACCCTtGAAGATTTTGTcGGCGATTGGCGCCAGACCGCGcGCTATAACCTcGATCAaGTGCTcGAACAaGGCGGCGTGAGCAGCCTGTTTCAGAACCTGGGCGTGAGCGTGACCCCGATTCAGCGCATTGTGCTGAGCGGCGAAAACGGCCTGAAAATTGATATTCATGTGATTATTCCGTATGAAGGCCTGAGCGGCGATCAGATGGGCCAGATTGAAAAAATTTTTAAAGTcGTaTATCCtGTcGATGATCATCATTTTAAAGTGATTCTGCATTATGGCACCCTGGTGATTGATGGCGTGACCCCGAACATGATTGATTATTTTGGCCGCCCGTATGAAGGCATTGCtGTtTTTGATGGCAAgAAgATTACCGTGACCGGCACCCTGTGGAACGGCAACAAAATTATTGATGAACGCCTGATTAACCCaGATGGCAGCCTGCTGTTTCGCGTGACCATTAACGGCGTGACCGGCTGGCGCCTGTGCGAACGCATTCTcGCaGACTACAAAGACCATGACGGTGATTATAAAGATCATGACATCGATTACAAGGATGACGATGACAAGTAAGGCCGCGACTCTAGAG

**Sc1 nLuc reporter**

*TAATACGACTCACTATAG*GGACATTTGCTTCTGACACAACTGTGTTCACTAGCAACCTCAAACAGACACCATGCAACAACAACAACAAGCTGCGGTGTGGAAGGAGTGGTCGGGTTGCGCAGCGCAACAACAACAACAAGGAAGCGGAGCTACTAACTTCAGCCTGCTGAAGCAaGCTGGAGACGTcGAaGAaAACCCTGGACCTGTGTTTACCCTtGAAGATTTTGTcGGCGATTGGCGCCAGACCGCGcGCTATAACCTcGATCAaGTGCTcGAACAaGGCGGCGTGAGCAGCCTGTTTCAGAACCTGGGCGTGAGCGTGACCCCGATTCAGCGCATTGTGCTGAGCGGCGAAAACGGCCTGAAAATTGATATTCATGTGATTATTCCGTATGAAGGCCTGAGCGGCGATCAGATGGGCCAGATTGAAAAAATTTTTAAAGTcGTaTATCCtGTcGATGATCATCATTTTAAAGTGATTCTGCATTATGGCACCCTGGTGATTGATGGCGTGACCCCGAACATGATTGATTATTTTGGCCGCCCGTATGAAGGCATTGCtGTtTTTGATGGCAAgAAgATTACCGTGACCGGCACCCTGTGGAACGGCAACAAAATTATTGATGAACGCCTGATTAACCCaGATGGCAGCCTGCTGTTTCGCGTGACCATTAACGGCGTGACCGGCTGGCGCCTGTGCGAACGCATTCTcGCaGACTACAAAGACCATGACGGTGATTATAAAGATCATGACATCGATTACAAGGATGACGATGACAAGTAAGGCCGCGACTCTAGAG

**Long 5ʹ UTR nLuc reporter**

*TAATACGACTCACTATAG*GGACATTTGCTTCTGACACAACTGTGTTCACTAGCAACCTCAAACAGACACCACATTTGCTTCTGACACAACTGTGTTCACTAGCAACCTCAAACAGACACCACATTTGCTTCTGACACAACTGTGTTCACTAGCAACCTCAAACAGACACCATGGGAAGCGGAGCTACTAACTTCAGCCTGCTGAAGCAaGCTGGAGACGTcGAaGAaAACCCTGGACCTGTGTTTACCCTtGAAGATTTTGTcGGCGATTGGCGCCAGACCGCGcGCTATAACCTcGATCAaGTGCTcGAACAaGGCGGCGTGAGCAGCCTGTTTCAGAACCTGGGCGTGAGCGTGACCCCGATTCAGCGCATTGTGCTGAGCGGCGAAAACGGCCTGAAAATTGATATTCATGTGATTATTCCGTATGAAGGCCTGAGCGGCGATCAGATGGGCCAGATTGAAAAAATTTTTAAAGTcGTaTATCCtGTcGATGATCATCATTTTAAAGTGATTCTGCATTATGGCACCCTGGTGATTGATGGCGTGACCCCGAACATGATTGATTATTTTGGCCGCCCGTATGAAGGCATTGCtGTtTTTGATGGCAAgAAgATTACCGTGACCGGCACCCTGTGGAACGGCAACAAAATTATTGATGAACGCCTGATTAACCCaGATGGCAGCCTGCTGTTTCGCGTGACCATTAACGGCGTGACCGGCTGGCGCCTGTGCGAACGCATTCTcGCaGACTACAAAGACCATGACGGTGATTATAAAGATCATGACATCGATTACAAGGATGACGATGACAAGTAAGGCCGCGACTCTAGAG

**G4-less mEGFP reporter**

*TAATACGACTCACTATAG*GGAGACCCAAGCTGGCTAGCGTTTAAACTTAAGCTTGGCAATCCGGTACTGTTGGTAAATAAGCCACCATGgtgagcaagggcgaggagctgttcaccggggtggtgcccatcctggtcgagctggacggcgacgtaaacggccacaagttcagcgtgtccggcgagggcgagggcgatgccacctacggcaagctgaccctgaagttcatctgcaccaccggcaagctgcccgtgccctggcccaccctcgtgaccaccctgacctacggcgtgcagtgcttcagccgctaccccgaccacatgaagcagcacgacttcttcaagtccgccatgcccgaaggctacgtccaggagcgcaccatcttcttcaaggacgacggcaactacaagacccgcgccgaggtgaagttcgagggcgacaccctggtgaaccgcatcgagctgaagggcatcgacttcaaggaggacggcaacatcctggggcacaagctggagtacaactacaacagccacaacgtctatatcatggccgacaagcagaagaacggcatcaaggtgaacttcaagatccgccacaacatcgaggacggcagcgtgcagctcgccgaccactaccagcagaacacccccatcggcgacggccccgtgctgctgcccgacaaccactacctgagcacccagtccaagctgagcaaagaccccaacgagaagcgcgatcacatggtcctgctggagttcgtgaccgccgccgggatcactctcggcatggacgagctgtacaagTAAGGCCGCGACTCTAGAG

**Recombinant Protein Coding Sequences**

Start codon

MBP

His6

NT-hFMRP (WT and mutant)

Stop codon

**His6-MBP**

atgggttcttctcaccatcaccatcaccatggttcttctatgaaaatcgaagaaggtaaactggtaatctggattaacggcgataaaggctataacggtctcgctgaagtcggtaagaaattcgagaaagataccggaattaaagtcaccgttgagcatccggataaactggaagagaaattcccacaggttgcggcaactggcgatggccctgacattatcttctgggcacacgaccgctttggtggctacgctcaatctggcctgttggctgaaatcaccccggacaaagcgttccaggacaagctgtatccgtttacctgggatgccgtacgttacaacggcaagctgattgcttacccgatcgctgttgaagcgttatcgctgatttataacaaagatctgctgccgaacccgccaaaaacctgggaagagatcccggcgctggataaagaactgaaagcgaaaggtaagagcgcgctgatgttcaacctgcaagaaccgtacttcacctggccgctgattgctgctgacgggggttatgcgttcaagtatgaaaacggcaagtacgacattaaagacgtgggcgtggataacgctggcgcgaaagcgggtctgaccttcctggttgacctgattaaaaacaaacacatgaatgcagacaccgattactccatcgcagaagctgcctttaataaaggcgaaacagcgatgaccatcaacggcccgtgggcatggtccaacatcgacaccagcaaagtgaattatggtgtaacggtactgccgaccttcaagggtcaaccatccaaaccgttcgttggcgtgctgagcgcaggtattaacgccgccagtccgaacaaagagctggcaaaagagttcctcgaaaactatctgctgactgatgaaggtctggaagcggttaataaagacaaaccgctgggtgccgtagcgctgaagtcttacgaggaagagttggcgaaagatccacgtattgccgccactatggaaaacgcccagaaaggtgaaatcatgccgaacatcccgcagatgtccgctttctggtatgccgtgcgtactgcggtgatcaacgccgccagcggtcgtcagactgtcgatgaagccctgaaagacgcgcagactaatgggatcgaggaaaacctgtacttccaatccaatattggaagtggataa

**MBP-(WT NT-hFMRP)-His6**

atgggttcttctatgaaaatcgaagaaggtaaactggtaatctggattaacggcgataaaggctataacggtctcgctgaagtcggtaagaaattcgagaaagataccggaattaaagtcaccgttgagcatccggataaactggaagagaaattcccacaggttgcggcaactggcgatggccctgacattatcttctgggcacacgaccgctttggtggctacgctcaatctggcctgttggctgaaatcaccccggacaaagcgttccaggacaagctgtatccgtttacctgggatgccgtacgttacaacggcaagctgattgcttacccgatcgctgttgaagcgttatcgctgatttataacaaagatctgctgccgaacccgccaaaaacctgggaagagatcccggcgctggataaagaactgaaagcgaaaggtaagagcgcgctgatgttcaacctgcaagaaccgtacttcacctggccgctgattgctgctgacgggggttatgcgttcaagtatgaaaacggcaagtacgacattaaagacgtgggcgtggataacgctggcgcgaaagcgggtctgaccttcctggttgacctgattaaaaacaaacacatgaatgcagacaccgattactccatcgcagaagctgcctttaataaaggcgaaacagcgatgaccatcaacggcccgtgggcatggtccaacatcgacaccagcaaagtgaattatggtgtaacggtactgccgaccttcaagggtcaaccatccaaaccgttcgttggcgtgctgagcgcaggtattaacgccgccagtccgaacaaagagctggcaaaagagttcctcgaaaactatctgctgactgatgaaggtctggaagcggttaataaagacaaaccgctgggtgccgtagcgctgaagtcttacgaggaagagttggcgaaagatccacgtattgccgccactatggaaaacgcccagaaaggtgaaatcatgccgaacatcccgcagatgtccgctttctggtatgccgtgcgtactgcggtgatcaacgccgccagcggtcgtcagactgtcgatgaagccctgaaagacgcgcagactaatgggatcgaggaaaacctgtacttccaatccaatGCACGCTTCCACGAACAATTTATTGTTCGCGAGGACCTGATGGGCTTGGCGATTGGCACCCACGGCGCGAACATTCAGCAGGCGCGTAAAGTCCCAGGTGTTACCGCTATTGATCTGGACGAAGACACGTGCACCTTTCATATTTATGGTGAAGACCAGGATGCTGTGAAAAAGGCGCGTTCCTTCCTGGAATTTGCGGAAGACGTGATCCAGGTTCCGCGTAACCTGGTTGGTAAAGTCATCGGCAAAAACGGTAAGTTAATCCAAGAGATCGTGGACAAAAGCGGGGTAGTTCGCGTTCGGATTGAAGCGGAAAATGAGAAGAACGTTCCGCAGGAGGAAGAAATTATGCCGCCAAATAGCCTGCCGAGCAACAACTCACGTGTCGGTCCGAACGCTCCGGAAGAGAAGAAGCACCTGGATATTAAAGAGAACAGCACCCATTTCAGCCAACCAAACTCCACTAAGGTGCAGCGTGTTTTGGTAGCCAGCTCCGTTGTTGCCGGTGAGTCGCAAAAGCCGGAACTGAAAGCGTGGCAGGGTATGGTGCCGTTCGTCTTTGTGGGCACCAAGGACAGCATCGCCAACGCAACGGTTCTGCTGGACTACCATCTGAATTACCTGAAAGAAGTCGATCAGCTTCGTTTGGAACGCTTGCAAATCGATGAGCAACTGCGCCAGATCGGTGCGAGCTCTCGCCCGtctCCGAACCGTACCGACAAAGAGAAGAGCTACGTGACCGACGACGGTCAAGGTATGGGCCGTGGCAGCCGTCCGTATCGTAATAGGGGACATGGCCGCCGTGGTCCGGGTTACACCTCAGGTACGAACTCTGAGGCGTCTAACGCCTCCGAAACCGAGTCGGATCATCGTGATGAGCTGAGCGACTGGTCACTGGCGCCGACCGAAGAGGAGCGCGAGAGCTTTCTGCGTCGCGGTGATGGTCGCCGCAGAGGCGGTGGCGGTCGTGGCCAGGGCGGCCGTGGCAGAGGCGGCGGTTTTAAAGGTAATGATGATCACAGCCGCACTGACAACCGTCCGCGTAATCCGCGTGAGGCGAAGGGCAGAACTACAGATGGTAGCTTGCAAATCCGTGTGGACTGTAATAACGAACGCAGCGTGCATACGAAAACCCTACAAAACACCAGTAGCGAGGGTAGCCGCCTTCGTACCGGTAAAGACCGCAACCAGAAGAAAGAGAAACCGGACAGCGTTGATGGCCAACAACCGTTGGTGAATGGTGTTCCGggttcttctcaccatcaccatcaccatTAA

**MBP-(I304N NT-hFMRP)-His6**

atgggttcttctatgaaaatcgaagaaggtaaactggtaatctggattaacggcgataaaggctataacggtctcgctgaagtcggtaagaaattcgagaaagataccggaattaaagtcaccgttgagcatccggataaactggaagagaaattcccacaggttgcggcaactggcgatggccctgacattatcttctgggcacacgaccgctttggtggctacgctcaatctggcctgttggctgaaatcaccccggacaaagcgttccaggacaagctgtatccgtttacctgggatgccgtacgttacaacggcaagctgattgcttacccgatcgctgttgaagcgttatcgctgatttataacaaagatctgctgccgaacccgccaaaaacctgggaagagatcccggcgctggataaagaactgaaagcgaaaggtaagagcgcgctgatgttcaacctgcaagaaccgtacttcacctggccgctgattgctgctgacgggggttatgcgttcaagtatgaaaacggcaagtacgacattaaagacgtgggcgtggataacgctggcgcgaaagcgggtctgaccttcctggttgacctgattaaaaacaaacacatgaatgcagacaccgattactccatcgcagaagctgcctttaataaaggcgaaacagcgatgaccatcaacggcccgtgggcatggtccaacatcgacaccagcaaagtgaattatggtgtaacggtactgccgaccttcaagggtcaaccatccaaaccgttcgttggcgtgctgagcgcaggtattaacgccgccagtccgaacaaagagctggcaaaagagttcctcgaaaactatctgctgactgatgaaggtctggaagcggttaataaagacaaaccgctgggtgccgtagcgctgaagtcttacgaggaagagttggcgaaagatccacgtattgccgccactatggaaaacgcccagaaaggtgaaatcatgccgaacatcccgcagatgtccgctttctggtatgccgtgcgtactgcggtgatcaacgccgccagcggtcgtcagactgtcgatgaagccctgaaagacgcgcagactaatgggatcgaggaaaacctgtacttccaatccaatGCACGCTTCCACGAACAATTTATTGTTCGCGAGGACCTGATGGGCTTGGCGATTGGCACCCACGGCGCGAACATTCAGCAGGCGCGTAAAGTCCCAGGTGTTACCGCTATTGATCTGGACGAAGACACGTGCACCTTTCATATTTATGGTGAAGACCAGGATGCTGTGAAAAAGGCGCGTTCCTTCCTGGAATTTGCGGAAGACGTGATCCAGGTTCCGCGTAACCTGGTTGGTAAAGTCATCGGCAAAAACGGTAAGTTAAACCAAGAGATCGTGGACAAAAGCGGGGTAGTTCGCGTTCGGATTGAAGCGGAAAATGAGAAGAACGTTCCGCAGGAGGAAGAAATTATGCCGCCAAATAGCCTGCCGAGCAACAACTCACGTGTCGGTCCGAACGCTCCGGAAGAGAAGAAGCACCTGGATATTAAAGAGAACAGCACCCATTTCAGCCAACCAAACTCCACTAAGGTGCAGCGTGTTTTGGTAGCCAGCTCCGTTGTTGCCGGTGAGTCGCAAAAGCCGGAACTGAAAGCGTGGCAGGGTATGGTGCCGTTCGTCTTTGTGGGCACCAAGGACAGCATCGCCAACGCAACGGTTCTGCTGGACTACCATCTGAATTACCTGAAAGAAGTCGATCAGCTTCGTTTGGAACGCTTGCAAATCGATGAGCAACTGCGCCAGATCGGTGCGAGCTCTCGCCCGtctCCGAACCGTACCGACAAAGAGAAGAGCTACGTGACCGACGACGGTCAAGGTATGGGCCGTGGCAGCCGTCCGTATCGTAATAGGGGACATGGCCGCCGTGGTCCGGGTTACACCTCAGGTACGAACTCTGAGGCGTCTAACGCCTCCGAAACCGAGTCGGATCATCGTGATGAGCTGAGCGACTGGTCACTGGCGCCGACCGAAGAGGAGCGCGAGAGCTTTCTGCGTCGCGGTGATGGTCGCCGCAGAGGCGGTGGCGGTCGTGGCCAGGGCGGCCGTGGCAGAGGCGGCGGTTTTAAAGGTAATGATGATCACAGCCGCACTGACAACCGTCCGCGTAATCCGCGTGAGGCGAAGGGCAGAACTACAGATGGTAGCTTGCAAATCCGTGTGGACTGTAATAACGAACGCAGCGTGCATACGAAAACCCTACAAAACACCAGTAGCGAGGGTAGCCGCCTTCGTACCGGTAAAGACCGCAACCAGAAGAAAGAGAAACCGGACAGCGTTGATGGCCAACAACCGTTGGTGAATGGTGTTCCGggttcttctcaccatcaccatcaccatTAA

**MBP-(ΔRGG+CTD NT-hFMRP)-His6**

atgggttcttctatgaaaatcgaagaaggtaaactggtaatctggattaacggcgataaaggctataacggtctcgctgaagtcggtaagaaattcgagaaagataccggaattaaagtcaccgttgagcatccggataaactggaagagaaattcccacaggttgcggcaactggcgatggccctgacattatcttctgggcacacgaccgctttggtggctacgctcaatctggcctgttggctgaaatcaccccggacaaagcgttccaggacaagctgtatccgtttacctgggatgccgtacgttacaacggcaagctgattgcttacccgatcgctgttgaagcgttatcgctgatttataacaaagatctgctgccgaacccgccaaaaacctgggaagagatcccggcgctggataaagaactgaaagcgaaaggtaagagcgcgctgatgttcaacctgcaagaaccgtacttcacctggccgctgattgctgctgacgggggttatgcgttcaagtatgaaaacggcaagtacgacattaaagacgtgggcgtggataacgctggcgcgaaagcgggtctgaccttcctggttgacctgattaaaaacaaacacatgaatgcagacaccgattactccatcgcagaagctgcctttaataaaggcgaaacagcgatgaccatcaacggcccgtgggcatggtccaacatcgacaccagcaaagtgaattatggtgtaacggtactgccgaccttcaagggtcaaccatccaaaccgttcgttggcgtgctgagcgcaggtattaacgccgccagtccgaacaaagagctggcaaaagagttcctcgaaaactatctgctgactgatgaaggtctggaagcggttaataaagacaaaccgctgggtgccgtagcgctgaagtcttacgaggaagagttggcgaaagatccacgtattgccgccactatggaaaacgcccagaaaggtgaaatcatgccgaacatcccgcagatgtccgctttctggtatgccgtgcgtactgcggtgatcaacgccgccagcggtcgtcagactgtcgatgaagccctgaaagacgcgcagactaatgggatcgaggaaaacctgtacttccaatccaatGCACGCTTCCACGAACAATTTATTGTTCGCGAGGACCTGATGGGCTTGGCGATTGGCACCCACGGCGCGAACATTCAGCAGGCGCGTAAAGTCCCAGGTGTTACCGCTATTGATCTGGACGAAGACACGTGCACCTTTCATATTTATGGTGAAGACCAGGATGCTGTGAAAAAGGCGCGTTCCTTCCTGGAATTTGCGGAAGACGTGATCCAGGTTCCGCGTAACCTGGTTGGTAAAGTCATCGGCAAAAACGGTAAGTTAATCCAAGAGATCGTGGACAAAAGCGGGGTAGTTCGCGTTCGGATTGAAGCGGAAAATGAGAAGAACGTTCCGCAGGAGGAAGAAATTATGCCGCCAAATAGCCTGCCGAGCAACAACTCACGTGTCGGTCCGAACGCTCCGGAAGAGAAGAAGCACCTGGATATTAAAGAGAACAGCACCCATTTCAGCCAACCAAACTCCACTAAGGTGCAGCGTGTTTTGGTAGCCAGCTCCGTTGTTGCCGGTGAGTCGCAAAAGCCGGAACTGAAAGCGTGGCAGGGTATGGTGCCGTTCGTCTTTGTGGGCACCAAGGACAGCATCGCCAACGCAACGGTTCTGCTGGACTACCATCTGAATTACCTGAAAGAAGTCGATCAGCTTCGTTTGGAACGCTTGCAAATCGATGAGCAACTGCGCCAGATCGGTGCGAGCTCTCGCCCGtctCCGAACCGTACCGACAAAGAGAAGAGCTACGTGACCGACGACGGTCAAGGTATGGGCCGTGGCAGCCGTCCGTATCGTAATAGGGGACATGGCCGCCGTGGTCCGGGTTACACCTCAGGTACGAACTCTGAGGCGTCTAACGCCTCCGAAACCGAGTCGGATCATCGTGATGAGCTGAGCGACTGGTCACTGGCGCCGACCGAAGAGGAGCGCGAGAGCTTTCTGCGTCGCGGTGATGGTCGCCGCAGAGGCGGTGGCGGGTCGTGGCCAGGGCGGCCGTGGCAGAGGCGGCGGTTTggttcttctcaccatcaccatcaccatTAA

**MBP-(NT-hFMRP ΔRGG+CTD complete)-His6**

atgggttcttctatgaaaatcgaagaaggtaaactggtaatctggattaacggcgataaaggctataacggtctcgctgaagtcggtaagaaattcgagaaagataccggaattaaagtcaccgttgagcatccggataaactggaagagaaattcccacaggttgcggcaactggcgatggccctgacattatcttctgggcacacgaccgctttggtggctacgctcaatctggcctgttggctgaaatcaccccggacaaagcgttccaggacaagctgtatccgtttacctgggatgccgtacgttacaacggcaagctgattgcttacccgatcgctgttgaagcgttatcgctgatttataacaaagatctgctgccgaacccgccaaaaacctgggaagagatcccggcgctggataaagaactgaaagcgaaaggtaagagcgcgctgatgttcaacctgcaagaaccgtacttcacctggccgctgattgctgctgacgggggttatgcgttcaagtatgaaaacggcaagtacgacattaaagacgtgggcgtggataacgctggcgcgaaagcgggtctgaccttcctggttgacctgattaaaaacaaacacatgaatgcagacaccgattactccatcgcagaagctgcctttaataaaggcgaaacagcgatgaccatcaacggcccgtgggcatggtccaacatcgacaccagcaaagtgaattatggtgtaacggtactgccgaccttcaagggtcaaccatccaaaccgttcgttggcgtgctgagcgcaggtattaacgccgccagtccgaacaaagagctggcaaaagagttcctcgaaaactatctgctgactgatgaaggtctggaagcggttaataaagacaaaccgctgggtgccgtagcgctgaagtcttacgaggaagagttggcgaaagatccacgtattgccgccactatggaaaacgcccagaaaggtgaaatcatgccgaacatcccgcagatgtccgctttctggtatgccgtgcgtactgcggtgatcaacgccgccagcggtcgtcagactgtcgatgaagccctgaaagacgcgcagactaatgggatcgaggaaaacctgtacttccaatccaatGCACGCTTCCACGAACAATTTATTGTTCGCGAGGACCTGATGGGCTTGGCGATTGGCACCCACGGCGCGAACATTCAGCAGGCGCGTAAAGTCCCAGGTGTTACCGCTATTGATCTGGACGAAGACACGTGCACCTTTCATATTTATGGTGAAGACCAGGATGCTGTGAAAAAGGCGCGTTCCTTCCTGGAATTTGCGGAAGACGTGATCCAGGTTCCGCGTAACCTGGTTGGTAAAGTCATCGGCAAAAACGGTAAGTTAATCCAAGAGATCGTGGACAAAAGCGGGGTAGTTCGCGTTCGGATTGAAGCGGAAAATGAGAAGAACGTTCCGCAGGAGGAAGAAATTATGCCGCCAAATAGCCTGCCGAGCAACAACTCACGTGTCGGTCCGAACGCTCCGGAAGAGAAGAAGCACCTGGATATTAAAGAGAACAGCACCCATTTCAGCCAACCAAACTCCACTAAGGTGCAGCGTGTTTTGGTAGCCAGCTCCGTTGTTGCCGGTGAGTCGCAAAAGCCGGAACTGAAAGCGTGGCAGGGTATGGTGCCGTTCGTCTTTGTGGGCACCAAGGACAGCATCGCCAACGCAACGGTTCTGCTGGACTACCATCTGAATTACCTGAAAGAAGTCGATCAGCTTCGTTTGGAACGCTTGCAAATCGATGAGCAACTGCGCCAGATCGGTGCGAGCTCTCGCCCGtctCCGAACCGTACCGACAAAGAGAAGAGCTACGTGACCGACGACGGTCAAGGTATGGGCCGTGGCAGCCGTCCGTATCGTAATAGGGGACATGGCCGCCGTGGTCCGGGTTACACCTCAGGTACGAACTCTGAGGCGTCTAACGCCTCCGAAACCGAGTCGGATCATCGTGATGAGCTGAGCGACTGGTCACTGGCGCCGACCGAAGAGGAGCGCGAGAGCTTTCTGggttcttctcaccatcaccatcaccatTAA

**MBP-(I304N & ΔRGG+CTD NT-hFMRP)-His6**

atgggttcttctatgaaaatcgaagaaggtaaactggtaatctggattaacggcgataaaggctataacggtctcgctgaagtcggtaagaaattcgagaaagataccggaattaaagtcaccgttgagcatccggataaactggaagagaaattcccacaggttgcggcaactggcgatggccctgacattatcttctgggcacacgaccgctttggtggctacgctcaatctggcctgttggctgaaatcaccccggacaaagcgttccaggacaagctgtatccgtttacctgggatgccgtacgttacaacggcaagctgattgcttacccgatcgctgttgaagcgttatcgctgatttataacaaagatctgctgccgaacccgccaaaaacctgggaagagatcccggcgctggataaagaactgaaagcgaaaggtaagagcgcgctgatgttcaacctgcaagaaccgtacttcacctggccgctgattgctgctgacgggggttatgcgttcaagtatgaaaacggcaagtacgacattaaagacgtgggcgtggataacgctggcgcgaaagcgggtctgaccttcctggttgacctgattaaaaacaaacacatgaatgcagacaccgattactccatcgcagaagctgcctttaataaaggcgaaacagcgatgaccatcaacggcccgtgggcatggtccaacatcgacaccagcaaagtgaattatggtgtaacggtactgccgaccttcaagggtcaaccatccaaaccgttcgttggcgtgctgagcgcaggtattaacgccgccagtccgaacaaagagctggcaaaagagttcctcgaaaactatctgctgactgatgaaggtctggaagcggttaataaagacaaaccgctgggtgccgtagcgctgaagtcttacgaggaagagttggcgaaagatccacgtattgccgccactatggaaaacgcccagaaaggtgaaatcatgccgaacatcccgcagatgtccgctttctggtatgccgtgcgtactgcggtgatcaacgccgccagcggtcgtcagactgtcgatgaagccctgaaagacgcgcagactaatgggatcgaggaaaacctgtacttccaatccaatGCAGCTTCCACGAACAATTTATTGTTCGCGAGGACCTGATGGGCTTGGCGATTGGCACCCACGGCGCGAACATTCAGCAGGCGCGTAAAGTCCCAGGTGTTACCGCTATTGATCTGGACGAAGACACGTGCACCTTTCATATTTATGGTGAAGACCAGGATGCTGTGAAAAAGGCGCGTTCCTTCCTGGAATTTGCGGAAGACGTGATCCAGGTTCCGCGTAACCTGGTTGGTAAAGTCATCGGCAAAAACGGTAAGTTAAACCAAGAGATCGTGGACAAAAGCGGGGTAGTTCGCGTTCGGATTGAAGCGGAAAATGAGAAGAACGTTCCGCAGGAGGAAGAAATTATGCCGCCAAATAGCCTGCCGAGCAACAACTCACGTGTCGGTCCGAACGCTCCGGAAGAGAAGAAGCACCTGGATATTAAAGAGAACAGCACCCATTTCAGCCAACCAAACTCCACTAAGGTGCAGCGTGTTTTGGTAGCCAGCTCCGTTGTTGCCGGTGAGTCGCAAAAGCCGGAACTGAAAGCGTGGCAGGGTATGGTGCCGTTCGTCTTTGTGGGCACCAAGGACAGCATCGCCAACGCAACGGTTCTGCTGGACTACCATCTGAATTACCTGAAAGAAGTCGATCAGCTTCGTTTGGAACGCTTGCAAATCGATGAGCAACTGCGCCAGATCGGTGCGAGCTCTCGCCCGtctCCGAACCGTACCGACAAAGAGAAGAGCTACGTGACCGACGACGGTCAAGGTATGGGCCGTGGCAGCCGTCCGTATCGTAATAGGGGACATGGCCGCCGTGGTCCGGGTTACACCTCAGGTACGAACTCTGAGGCGTCTAACGCCTCCGAAACCGAGTCGGATCATCGTGATGAGCTGAGCGACTGGTCACTGGCGCCGACCGAAGAGGAGCGCGAGAGCTTTCTGCGTCGCGGTGATGGTCGCCGCAGAGGCGGTGGCGGGTCGTGGCCAGGGCGGCCGTGGCAGAGGCGGCGGTTTggttcttctcaccatcaccatcaccatTAA

**MBP-(NT-hFMRP Δ54)-His6**

atgggttcttctatgaaaatcgaagaaggtaaactggtaatctggattaacggcgataaaggctataacggtctcgctgaagtcggtaagaaattcgagaaagataccggaattaaagtcaccgttgagcatccggataaactggaagagaaattcccacaggttgcggcaactggcgatggccctgacattatcttctgggcacacgaccgctttggtggctacgctcaatctggcctgttggctgaaatcaccccggacaaagcgttccaggacaagctgtatccgtttacctgggatgccgtacgttacaacggcaagctgattgcttacccgatcgctgttgaagcgttatcgctgatttataacaaagatctgctgccgaacccgccaaaaacctgggaagagatcccggcgctggataaagaactgaaagcgaaaggtaagagcgcgctgatgttcaacctgcaagaaccgtacttcacctggccgctgattgctgctgacgggggttatgcgttcaagtatgaaaacggcaagtacgacattaaagacgtgggcgtggataacgctggcgcgaaagcgggtctgaccttcctggttgacctgattaaaaacaaacacatgaatgcagacaccgattactccatcgcagaagctgcctttaataaaggcgaaacagcgatgaccatcaacggcccgtgggcatggtccaacatcgacaccagcaaagtgaattatggtgtaacggtactgccgaccttcaagggtcaaccatccaaaccgttcgttggcgtgctgagcgcaggtattaacgccgccagtccgaacaaagagctggcaaaagagttcctcgaaaactatctgctgactgatgaaggtctggaagcggttaataaagacaaaccgctgggtgccgtagcgctgaagtcttacgaggaagagttggcgaaagatccacgtattgccgccactatggaaaacgcccagaaaggtgaaatcatgccgaacatcccgcagatgtccgctttctggtatgccgtgcgtactgcggtgatcaacgccgccagcggtcgtcagactgtcgatgaagccctgaaagacgcgcagactaatgggatcgaggaaaacctgtacttccaatccaatGCACGCTTCCACGAACAATTTATTGTTCGCGAGGACCTGATGGGCTTGGCGATTGGCACCCACGGCGCGAACATTCAGCAGGCGCGTAAAGTCCCAGGTGTTACCGCTATTGATCTGGACGAAGACACGTGCACCTTTCATATTTATGGTGAAGACCAGGATGCTGTGAAAAAGGCGCGTTCCTTCCTGGAATTTGCGGAAGACGTGATCCAGGTTCCGCGTAACCTGGTTGGTAAAGTCATCGGCAAAAACGGTAAGTTAATCCAAGAGATCGTGGACAAAAGCGGGGTAGTTCGCGTTCGGATTGAAGCGGAAAATGAGAAGAACGTTCCGCAGGAGGAAGAAATTATGCCGCCAAATAGCCTGCCGAGCAACAACTCACGTGTCGGTCCGAACGCTCCGGAAGAGAAGAAGCACCTGGATATTAAAGAGAACAGCACCCATTTCAGCCAACCAAACTCCACTAAGGTGCAGCGTGTTTTGGTAGCCAGCTCCGTTGTTGCCGGTGAGTCGCAAAAGCCGGAACTGAAAGCGTGGCAGGGTATGGTGCCGTTCGTCTTTGTGGGCACCAAGGACAGCATCGCCAACGCAACGGTTCTGCTGGACTACCATCTGAATTACCTGAAAGAAGTCGATCAGCTTCGTTTGGAACGCTTGCAAATCGATGAGCAACTGCGCCAGATCGGTGCGAGCTCTCGCCCGtctCCGAACCGTACCGACAAAGAGAAGAGCTACGTGACCGACGACGGTCAAGGTATGGGCCGTGGCAGCCGTCCGTATCGTAATAGGGGACATGGCCGCCGTGGTCCGGGTTACACCTCAGGTACGAACTCTGAGGCGTCTAACGCCTCCGAAACCGAGTCGGATCATCGTGATGAGCTGAGCGACTGGTCACTGGCGCCGACCGAAGAGGAGCGCGAGAGCTTTCTGCGTCGCGGTGATGGTCGCCGCAGAGGCGGTGGCGGTCGTGGCCAGGGCGGCCGTGGCAGAGGCGGCGGTTTTAAAGGTAATGATGATCACAGCCGCACTGACAACCGTCCGCGTAATCCGCGTGAGGCGAAGGGCAGAACTACAGATGGTAGCTTGggttcttctcaccatcaccatcaccatTAA

**MBP-(NT-hFMRP Δ55)-His6**

atgggttcttctatgaaaatcgaagaaggtaaactggtaatctggattaacggcgataaaggctataacggtctcgctgaagtcggtaagaaattcgagaaagataccggaattaaagtcaccgttgagcatccggataaactggaagagaaattcccacaggttgcggcaactggcgatggccctgacattatcttctgggcacacgaccgctttggtggctacgctcaatctggcctgttggctgaaatcaccccggacaaagcgttccaggacaagctgtatccgtttacctgggatgccgtacgttacaacggcaagctgattgcttacccgatcgctgttgaagcgttatcgctgatttataacaaagatctgctgccgaacccgccaaaaacctgggaagagatcccggcgctggataaagaactgaaagcgaaaggtaagagcgcgctgatgttcaacctgcaagaaccgtacttcacctggccgctgattgctgctgacgggggttatgcgttcaagtatgaaaacggcaagtacgacattaaagacgtgggcgtggataacgctggcgcgaaagcgggtctgaccttcctggttgacctgattaaaaacaaacacatgaatgcagacaccgattactccatcgcagaagctgcctttaataaaggcgaaacagcgatgaccatcaacggcccgtgggcatggtccaacatcgacaccagcaaagtgaattatggtgtaacggtactgccgaccttcaagggtcaaccatccaaaccgttcgttggcgtgctgagcgcaggtattaacgccgccagtccgaacaaagagctggcaaaagagttcctcgaaaactatctgctgactgatgaaggtctggaagcggttaataaagacaaaccgctgggtgccgtagcgctgaagtcttacgaggaagagttggcgaaagatccacgtattgccgccactatggaaaacgcccagaaaggtgaaatcatgccgaacatcccgcagatgtccgctttctggtatgccgtgcgtactgcggtgatcaacgccgccagcggtcgtcagactgtcgatgaagccctgaaagacgcgcagactaatgggatcgaggaaaacctgtacttccaatccaatGCACGCTTCCACGAACAATTTATTGTTCGCGAGGACCTGATGGGCTTGGCGATTGGCACCCACGGCGCGAACATTCAGCAGGCGCGTAAAGTCCCAGGTGTTACCGCTATTGATCTGGACGAAGACACGTGCACCTTTCATATTTATGGTGAAGACCAGGATGCTGTGAAAAAGGCGCGTTCCTTCCTGGAATTTGCGGAAGACGTGATCCAGGTTCCGCGTAACCTGGTTGGTAAAGTCATCGGCAAAAACGGTAAGTTAATCCAAGAGATCGTGGACAAAAGCGGGGTAGTTCGCGTTCGGATTGAAGCGGAAAATGAGAAGAACGTTCCGCAGGAGGAAGAAATTATGCCGCCAAATAGCCTGCCGAGCAACAACTCACGTGTCGGTCCGAACGCTCCGGAAGAGAAGAAGCACCTGGATATTAAAGAGAACAGCACCCATTTCAGCCAACCAAACTCCACTAAGGTGCAGCGTGTTTTGGTAGCCAGCTCCGTTGTTGCCGGTGAGTCGCAAAAGCCGGAACTGAAAGCGTGGCAGGGTATGGTGCCGTTCGTCTTTGTGGGCACCAAGGACAGCATCGCCAACGCAACGGTTCTGCTGGACTACCATCTGAATTACCTGAAAGAAGTCGATCAGCTTCGTTTGGAACGCTTGCAAATCGATGAGCAACTGCGCCAGATCGGTGCGAGCTCTCGCCCGtctCCGAACCGTACCGACAAAGAGAAGAGCTACGTGACCGACGACGGTCAAGGTATGGGCCGTGGCAGCCGTCCGTATCGTAATAGGGGACATGGCCGCCGTGGTCCGGGTTACACCTCAGGTACGAACTCTGAGGCGTCTAACGCCTCCGAAACCGAGTCGGATCATCGTGATGAGCTGAGCGACTGGTCACTGGCGCCGACCGAAGAGGAGCGCGAGAGCTTTCTGCGTCGCGGTGATGGTCGCCGCAGAGGCGGTGGCGGTCGTGGCCAGGGCGGCCGTGGCAGAGGCGGCGGTTTTAAAGGTAATGATGATCACAGCCGCACTGACAACCGTCCGCGTAATCCGCGTGAGGCGAAGGGCAGAACTACAGATGGTAGCggttcttctcaccatcaccatcaccatTAA

**MBP-(RGG+CTD)-His6**

atgggttcttctatgaaaatcgaagaaggtaaactggtaatctggattaacggcgataaaggctataacggtctcgctgaagtcggtaagaaattcgagaaagataccggaattaaagtcaccgttgagcatccggataaactggaagagaaattcccacaggttgcggcaactggcgatggccctgacattatcttctgggcacacgaccgctttggtggctacgctcaatctggcctgttggctgaaatcaccccggacaaagcgttccaggacaagctgtatccgtttacctgggatgccgtacgttacaacggcaagctgattgcttacccgatcgctgttgaagcgttatcgctgatttataacaaagatctgctgccgaacccgccaaaaacctgggaagagatcccggcgctggataaagaactgaaagcgaaaggtaagagcgcgctgatgttcaacctgcaagaaccgtacttcacctggccgctgattgctgctgacgggggttatgcgttcaagtatgaaaacggcaagtacgacattaaagacgtgggcgtggataacgctggcgcgaaagcgggtctgaccttcctggttgacctgattaaaaacaaacacatgaatgcagacaccgattactccatcgcagaagctgcctttaataaaggcgaaacagcgatgaccatcaacggcccgtgggcatggtccaacatcgacaccagcaaagtgaattatggtgtaacggtactgccgaccttcaagggtcaaccatccaaaccgttcgttggcgtgctgagcgcaggtattaacgccgccagtccgaacaaagagctggcaaaagagttcctcgaaaactatctgctgactgatgaaggtctggaagcggttaataaagacaaaccgctgggtgccgtagcgctgaagtcttacgaggaagagttggcgaaagatccacgtattgccgccactatggaaaacgcccagaaaggtgaaatcatgccgaacatcccgcagatgtccgctttctggtatgccgtgcgtactgcggtgatcaacgccgccagcggtcgtcagactgtcgatgaagccctgaaagacgcgcagactaatgggatcgaggaaaacctgtacttccaatccaatGCACGTCGCGGTGATGGTCGCCGCAGAGGCGGTGGCGGTCGTGGCCAGGGCGGCCGTGGCAGAGGCGGCGGTTTTAAAGGTAATGATGATCACAGCCGCACTGACAACCGTCCGCGTAATCCGCGTGAGGCGAAGGGCAGAACTACAGATGGTAGCTTGCAAATCCGTGTGGACTGTAATAACGAACGCAGCGTGCATACGAAAACCCTACAAAACACCAGTAGCGAGGGTAGCCGCCTTCGTACCGGTAAAGACCGCAACCAGAAGAAAGAGAAACCGGACAGCGTTGATGGCCAACAACCGTTGGTGAATGGTGTTCCGggttcttctcaccatcaccatcaccatTAA

**MBP-(RGG)-His6**

atgggttcttctatgaaaatcgaagaaggtaaactggtaatctggattaacggcgataaaggctataacggtctcgctgaagtcggtaagaaattcgagaaagataccggaattaaagtcaccgttgagcatccggataaactggaagagaaattcccacaggttgcggcaactggcgatggccctgacattatcttctgggcacacgaccgctttggtggctacgctcaatctggcctgttggctgaaatcaccccggacaaagcgttccaggacaagctgtatccgtttacctgggatgccgtacgttacaacggcaagctgattgcttacccgatcgctgttgaagcgttatcgctgatttataacaaagatctgctgccgaacccgccaaaaacctgggaagagatcccggcgctggataaagaactgaaagcgaaaggtaagagcgcgctgatgttcaacctgcaagaaccgtacttcacctggccgctgattgctgctgacgggggttatgcgttcaagtatgaaaacggcaagtacgacattaaagacgtgggcgtggataacgctggcgcgaaagcgggtctgaccttcctggttgacctgattaaaaacaaacacatgaatgcagacaccgattactccatcgcagaagctgcctttaataaaggcgaaacagcgatgaccatcaacggcccgtgggcatggtccaacatcgacaccagcaaagtgaattatggtgtaacggtactgccgaccttcaagggtcaaccatccaaaccgttcgttggcgtgctgagcgcaggtattaacgccgccagtccgaacaaagagctggcaaaagagttcctcgaaaactatctgctgactgatgaaggtctggaagcggttaataaagacaaaccgctgggtgccgtagcgctgaagtcttacgaggaagagttggcgaaagatccacgtattgccgccactatggaaaacgcccagaaaggtgaaatcatgccgaacatcccgcagatgtccgctttctggtatgccgtgcgtactgcggtgatcaacgccgccagcggtcgtcagactgtcgatgaagccctgaaagacgcgcagactaatgggatcgaggaaaacctgtacttccaatccaatGCACGTCGCGGTGATGGTCGCCGCAGAGGCGGTGGCGGTCGTGGCCAGGGCGGCCGTGGCAGAGGCGGCGGTTTTAAAGGTAATGATGATCACAGCCGCggttcttctcaccatcaccatcaccatTAA

**MBP-(CTD)-His6**

atgggttcttctatgaaaatcgaagaaggtaaactggtaatctggattaacggcgataaaggctataacggtctcgctgaagtcggtaagaaattcgagaaagataccggaattaaagtcaccgttgagcatccggataaactggaagagaaattcccacaggttgcggcaactggcgatggccctgacattatcttctgggcacacgaccgctttggtggctacgctcaatctggcctgttggctgaaatcaccccggacaaagcgttccaggacaagctgtatccgtttacctgggatgccgtacgttacaacggcaagctgattgcttacccgatcgctgttgaagcgttatcgctgatttataacaaagatctgctgccgaacccgccaaaaacctgggaagagatcccggcgctggataaagaactgaaagcgaaaggtaagagcgcgctgatgttcaacctgcaagaaccgtacttcacctggccgctgattgctgctgacgggggttatgcgttcaagtatgaaaacggcaagtacgacattaaagacgtgggcgtggataacgctggcgcgaaagcgggtctgaccttcctggttgacctgattaaaaacaaacacatgaatgcagacaccgattactccatcgcagaagctgcctttaataaaggcgaaacagcgatgaccatcaacggcccgtgggcatggtccaacatcgacaccagcaaagtgaattatggtgtaacggtactgccgaccttcaagggtcaaccatccaaaccgttcgttggcgtgctgagcgcaggtattaacgccgccagtccgaacaaagagctggcaaaagagttcctcgaaaactatctgctgactgatgaaggtctggaagcggttaataaagacaaaccgctgggtgccgtagcgctgaagtcttacgaggaagagttggcgaaagatccacgtattgccgccactatggaaaacgcccagaaaggtgaaatcatgccgaacatcccgcagatgtccgctttctggtatgccgtgcgtactgcggtgatcaacgccgccagcggtcgtcagactgtcgatgaagccctgaaagacgcgcagactaatgggatcgaggaaaacctgtacttccaatccaatGCAACTGACAACCGTCCGCGTAATCCGCGTGAGGCGAAGGGCAGAACTACAGATGGTAGCTTGCAAATCCGTGTGGACTGTAATAACGAACGCAGCGTGCATACGAAAACCCTACAAAACACCAGTAGCGAGGGTAGCCGCCTTCGTACCGGTAAAGACCGCAACCAGAAGAAAGAGAAACCGGACAGCGTTGATGGCCAACAACCGTTGGTGAATGGTGTTCCGggttcttctcaccatcaccatcaccatTAA

**MBP-(RGG+CTD Δ19)-His6**

atgggttcttctatgaaaatcgaagaaggtaaactggtaatctggattaacggcgataaaggctataacggtctcgctgaagtcggtaagaaattcgagaaagataccggaattaaagtcaccgttgagcatccggataaactggaagagaaattcccacaggttgcggcaactggcgatggccctgacattatcttctgggcacacgaccgctttggtggctacgctcaatctggcctgttggctgaaatcaccccggacaaagcgttccaggacaagctgtatccgtttacctgggatgccgtacgttacaacggcaagctgattgcttacccgatcgctgttgaagcgttatcgctgatttataacaaagatctgctgccgaacccgccaaaaacctgggaagagatcccggcgctggataaagaactgaaagcgaaaggtaagagcgcgctgatgttcaacctgcaagaaccgtacttcacctggccgctgattgctgctgacgggggttatgcgttcaagtatgaaaacggcaagtacgacattaaagacgtgggcgtggataacgctggcgcgaaagcgggtctgaccttcctggttgacctgattaaaaacaaacacatgaatgcagacaccgattactccatcgcagaagctgcctttaataaaggcgaaacagcgatgaccatcaacggcccgtgggcatggtccaacatcgacaccagcaaagtgaattatggtgtaacggtactgccgaccttcaagggtcaaccatccaaaccgttcgttggcgtgctgagcgcaggtattaacgccgccagtccgaacaaagagctggcaaaagagttcctcgaaaactatctgctgactgatgaaggtctggaagcggttaataaagacaaaccgctgggtgccgtagcgctgaagtcttacgaggaagagttggcgaaagatccacgtattgccgccactatggaaaacgcccagaaaggtgaaatcatgccgaacatcccgcagatgtccgctttctggtatgccgtgcgtactgcggtgatcaacgccgccagcggtcgtcagactgtcgatgaagccctgaaagacgcgcagactaatgggatcgaggaaaacctgtacttccaatccaatGCACGTCGCGGTGATGGTCGCCGCAGAGGCGGTGGCGGTCGTGGCCAGGGCGGCCGTGGCAGAGGCGGCGGTTTTAAAGGTAATGATGATCACAGCCGCACTGACAACCGTCCGCGTAATCCGCGTGAGGCGAAGGGCAGAACTACAGATGGTAGCTTGCAAATCCGTGTGGACTGTAATAACGAACGCAGCGTGCATACGAAAACCCTACAAAACACCAGTAGCGAGGGTAGCCGCCTTCGTACCGGTAAAGACCGCAACCAGggttcttctcaccatcaccatcaccatTAA

**MBP-(RGG+CTD Δ54)-His6**

atgggttcttctatgaaaatcgaagaaggtaaactggtaatctggattaacggcgataaaggctataacggtctcgctgaagtcggtaagaaattcgagaaagataccggaattaaagtcaccgttgagcatccggataaactggaagagaaattcccacaggttgcggcaactggcgatggccctgacattatcttctgggcacacgaccgctttggtggctacgctcaatctggcctgttggctgaaatcaccccggacaaagcgttccaggacaagctgtatccgtttacctgggatgccgtacgttacaacggcaagctgattgcttacccgatcgctgttgaagcgttatcgctgatttataacaaagatctgctgccgaacccgccaaaaacctgggaagagatcccggcgctggataaagaactgaaagcgaaaggtaagagcgcgctgatgttcaacctgcaagaaccgtacttcacctggccgctgattgctgctgacgggggttatgcgttcaagtatgaaaacggcaagtacgacattaaagacgtgggcgtggataacgctggcgcgaaagcgggtctgaccttcctggttgacctgattaaaaacaaacacatgaatgcagacaccgattactccatcgcagaagctgcctttaataaaggcgaaacagcgatgaccatcaacggcccgtgggcatggtccaacatcgacaccagcaaagtgaattatggtgtaacggtactgccgaccttcaagggtcaaccatccaaaccgttcgttggcgtgctgagcgcaggtattaacgccgccagtccgaacaaagagctggcaaaagagttcctcgaaaactatctgctgactgatgaaggtctggaagcggttaataaagacaaaccgctgggtgccgtagcgctgaagtcttacgaggaagagttggcgaaagatccacgtattgccgccactatggaaaacgcccagaaaggtgaaatcatgccgaacatcccgcagatgtccgctttctggtatgccgtgcgtactgcggtgatcaacgccgccagcggtcgtcagactgtcgatgaagccctgaaagacgcgcagactaatgggatcgaggaaaacctgtacttccaatccaatGCACGTCGCGGTGATGGTCGCCGCAGAGGCGGTGGCGGTCGTGGCCAGGGCGGCCGTGGCAGAGGCGGCGGTTTTAAAGGTAATGATGATCACAGCCGCACTGACAACCGTCCGCGTAATCCGCGTGAGGCGAAGGGCAGAACTACAGATGGTAGCTTGggttcttctcaccatcaccatcaccatTAA

**MBP-(RGG+CTD Δ55)-His6**

atgggttcttctatgaaaatcgaagaaggtaaactggtaatctggattaacggcgataaaggctataacggtctcgctgaagtcggtaagaaattcgagaaagataccggaattaaagtcaccgttgagcatccggataaactggaagagaaattcccacaggttgcggcaactggcgatggccctgacattatcttctgggcacacgaccgctttggtggctacgctcaatctggcctgttggctgaaatcaccccggacaaagcgttccaggacaagctgtatccgtttacctgggatgccgtacgttacaacggcaagctgattgcttacccgatcgctgttgaagcgttatcgctgatttataacaaagatctgctgccgaacccgccaaaaacctgggaagagatcccggcgctggataaagaactgaaagcgaaaggtaagagcgcgctgatgttcaacctgcaagaaccgtacttcacctggccgctgattgctgctgacgggggttatgcgttcaagtatgaaaacggcaagtacgacattaaagacgtgggcgtggataacgctggcgcgaaagcgggtctgaccttcctggttgacctgattaaaaacaaacacatgaatgcagacaccgattactccatcgcagaagctgcctttaataaaggcgaaacagcgatgaccatcaacggcccgtgggcatggtccaacatcgacaccagcaaagtgaattatggtgtaacggtactgccgaccttcaagggtcaaccatccaaaccgttcgttggcgtgctgagcgcaggtattaacgccgccagtccgaacaaagagctggcaaaagagttcctcgaaaactatctgctgactgatgaaggtctggaagcggttaataaagacaaaccgctgggtgccgtagcgctgaagtcttacgaggaagagttggcgaaagatccacgtattgccgccactatggaaaacgcccagaaaggtgaaatcatgccgaacatcccgcagatgtccgctttctggtatgccgtgcgtactgcggtgatcaacgccgccagcggtcgtcagactgtcgatgaagccctgaaagacgcgcagactaatgggatcgaggaaaacctgtacttccaatccaatGCACGTCGCGGTGATGGTCGCCGCAGAGGCGGTGGCGGTCGTGGCCAGGGCGGCCGTGGCAGAGGCGGCGGTTTTAAAGGTAATGATGATCACAGCCGCACTGACAACCGTCCGCGTAATCCGCGTGAGGCGAAGGGCAGAACTACAGATGGTAGCggttcttctcaccatcaccatcaccatTAA

**MBP-(RGG+CTD Δ62)-His6**

atgggttcttctatgaaaatcgaagaaggtaaactggtaatctggattaacggcgataaaggctataacggtctcgctgaagtcggtaagaaattcgagaaagataccggaattaaagtcaccgttgagcatccggataaactggaagagaaattcccacaggttgcggcaactggcgatggccctgacattatcttctgggcacacgaccgctttggtggctacgctcaatctggcctgttggctgaaatcaccccggacaaagcgttccaggacaagctgtatccgtttacctgggatgccgtacgttacaacggcaagctgattgcttacccgatcgctgttgaagcgttatcgctgatttataacaaagatctgctgccgaacccgccaaaaacctgggaagagatcccggcgctggataaagaactgaaagcgaaaggtaagagcgcgctgatgttcaacctgcaagaaccgtacttcacctggccgctgattgctgctgacgggggttatgcgttcaagtatgaaaacggcaagtacgacattaaagacgtgggcgtggataacgctggcgcgaaagcgggtctgaccttcctggttgacctgattaaaaacaaacacatgaatgcagacaccgattactccatcgcagaagctgcctttaataaaggcgaaacagcgatgaccatcaacggcccgtgggcatggtccaacatcgacaccagcaaagtgaattatggtgtaacggtactgccgaccttcaagggtcaaccatccaaaccgttcgttggcgtgctgagcgcaggtattaacgccgccagtccgaacaaagagctggcaaaagagttcctcgaaaactatctgctgactgatgaaggtctggaagcggttaataaagacaaaccgctgggtgccgtagcgctgaagtcttacgaggaagagttggcgaaagatccacgtattgccgccactatggaaaacgcccagaaaggtgaaatcatgccgaacatcccgcagatgtccgctttctggtatgccgtgcgtactgcggtgatcaacgccgccagcggtcgtcagactgtcgatgaagccctgaaagacgcgcagactaatgggatcgaggaaaacctgtacttccaatccaatGCACGTCGCGGTGATGGTCGCCGCAGAGGCGGTGGCGGTCGTGGCCAGGGCGGCCGTGGCAGAGGCGGCGGTTTTAAAGGTAATGATGATCACAGCCGCACTGACAACCGTCCGCGTAATCCGCGTGAGGCGAAGggttcttctcaccatcaccatcaccatTAA
